# Unravelling the link between phthalate exposure and endometriosis in humans: a systematic review and meta-analysis of the literature

**DOI:** 10.1007/s10815-021-02265-3

**Published:** 2021-07-06

**Authors:** Alessandro Conforti, Luigi Carbone, Vittorio Simeon, Paolo Chiodini, Vincenzo Marrone, Francesca Bagnulo, Federica Cariati, Ida Strina, Carlo Alviggi

**Affiliations:** 1 Department of Neuroscience, Reproductive Science and Odontostomatology, Federico II University, Via Sergio Pansini no. 5, 80131 Naples, Italy; 2 Medical Statistic Unit, Luigi Vanvitelli University, Naples, Italy

**Keywords:** Endometriosis, Phthalates, Phthalate esters, Endocrine disruptor chemicals, Urine analysis, Environmental disease

## Abstract

**Purpose:**

Endometriosis is a chronic debilitating inflammatory pathology which interests females in their reproductive age. Its pathogenesis has not yet been clearly defined. Recent evidence linked chemical agents as endocrine-disrupting chemicals to endometriosis. Phthalates are a widely used class of such compounds. This study aimed to summarize the current literature evaluating the link between exposure to phthalates and occurrence of endometriosis.

**Methods:**

A systematic review of literature and meta-analysis has been carried out following PRISMA guidelines to assess such link. Fourteen studies have been included in the review. Risk of bias has been assessed through the Newcastle Ottawa Scale.

**Results:**

We observed association between endometriosis and increased urinary levels of MBP/MnBP, MEOHP, and MEHHP, but not for others. Blood-derived analysis showed statistically significant link between endometriosis and BBP, DEHP, DnBP, and MEHP.

**Conclusion:**

Given the wide heterogeneity of included studies, results should be taken with caution. Further studies with more rigorous methodology are encouraged to unravel the true link between this class of toxic compounds and manifestation of endometriosis.

**Supplementary Information:**

The online version contains supplementary material available at 10.1007/s10815-021-02265-3.

## Introduction

Endometriosis is one of the most frequent gynecological diseases, affecting 6–10% of women of reproductive age [1]. Endometriosis is characterized by the presence of endometrial-like tissue outside the uterus that in turn provokes chronic pelvic inflammation [2–5].

In rare cases, endometriosis involves extra-pelvic organs such as the gastrointestinal or urinary tracts [6]. Clinical manifestations are widely variable among patients and can include dysmenorrhea, chronic pelvic pain, dyspareunia, infertility, and bowel and/or urinary disorders when these organs are involved [7, 8]. Several pathogenic hypotheses for endometriosis have been proposed to date [9, 10]. One of the most renowned theories proposed by Sampson in the 1920s is that endometriosis occurs as the result of the retrograde menstruation of endometrial tissue from the fallopian tubes into the peritoneal cavity [11, 12]. A more recent “embryogenic” theory suggests that endometriosis is related to the localization of embryologic endometrial tissue outside the uterine cavity during organogenesis [13, 14]. Endometriotic tissue is characterized by the disruption of estrogen and progesterone signaling resulting in estrogen dominance and progesterone resistance [15]. This disruption can be induced by endocrine-disrupting chemicals (EDCs) that bind and regulate hormonal receptors with agonist or antagonist activity. The Endocrine Society defines EDCs as “an exogenous chemical, or mixture of chemicals, that interferes with any aspect of hormone action” [16]. Phthalates are a class of known EDCs that are posited to exert estrogen-like activity and have been associated with endometriosis in humans [17, 18]. Phthalates are synthetic alkyl diesters of phthalic acid that undergo phase I and phase II metabolism and are mainly eliminated by urine in the form of mono-esters [17]. The T_max_ of phthalate compounds ranges from 2 to 24 h [19]. Moreover, phthalate metabolites have been identified in the peripheral circulation and can be stored in fat tissue or secreted in breast milk [20–22]. In a recent multicenter study, we demonstrated that phthalates accumulated in the serum and follicular fluid of women who underwent assisted reproductive techniques [23].

Phthalates are largely used in consumer products industry as solvents, additives, and plasticizers. [24]. They confer plasticity to rigid materials such as polyvinyl chloride and other polymers, but also lubricate, act as solvents, and otherwise provide favorable characteristics to products [25]. Their features may also differ, according to intrinsic properties of the specific phthalate, depending on chemical structure [24, 25]. In detail, apart from plastics, phthalates are commonly used in the manufacturing process of cosmetics (nail polish), body lotions, hair care products (shampoo, hair spray), and paints [17, 24, 25]. Accordingly, estimates of phthalate exposure are higher among women than men [26]. Phthalates are also used to produce medical devices and medications such as didanosine, omeprazole, and theophylline, and phthalate metabolites have been detected in patients taking these medications [17, 27]. Exposure to phthalates is facilitated by the fact that not being chemically attached in a stable manner to the other chemical constituents of the various industrial products, they can easily disperse into the environment [17, 24, 25, 28]. Therefore, given the widespread and various use, routes of exposure include ingestion, inhalation, dermal absorption, and intravenous injection [17, 25]. MBP (mono-*n*-butyl phthalate) and MiBP (mono-*iso*-butyl phthalate) as well as major DEHP (di-[2-ethyl-hexyl] phthalate) metabolites such as MEHHP (mono-[2-ethyl-5-hydroxyhexyl] phthalate) and MEOHP (mono-[2 ethyl-5-oxohexyl] phthalate) are the most common phthalate metabolites detected in humans [29, 30]. Evidence suggests that, similar to their parent compounds, phthalate metabolites are also bioactive: in a study by Wang et al. [31], MEHP (mono-[2-ethylhexyl] phthalate) influenced prostaglandin secretion from bovine endometrial stromal cells. Phthalate metabolites may also interact directly with androgen and estrogen receptors [32]. With respect to female reproductive function, phthalates have been implicated in menstrual cycle pathophysiology and polycystic ovary syndrome [33]. DEHP has been evaluated for reproductive effects in humans and in animal models [34, 35] as well as other phthalates that produce biological consequences for placental and gamete functions [17]. Specifically, exposure to phthalates was associated with changes to placental cell DNA methylation patterns and genomic imprinting [36], and in another study altered transcriptomic activity in oocytes and subsequent blastocysts [37]. Given the widespread use of phthalates and their proposed impact on various aspects of health and reproduction, several countries have planned and implemented epidemiological biomonitoring studies to quantify phthalate levels in humans [38]. A 2011 Chinese study described MBP and MiBP as the major metabolites identified among their study population [29], while MBP, MEP (mono-ethyl phthalate), and major DEHP metabolites such as MEHHP and MEOHP were reported in a German population in 2003 [30]. Since these studies, strict campaigns have been enacted to reduce or limit the use of phthalates in these countries. The exact role of phthalate exposure in endometriosis remains unclear. Several studies have demonstrated that phthalates can bind estrogen receptors, induce oxidative stress, and activate metabolic pathways associated with the pathogenesis of endometriosis [21, 39]. Yet, investigations of a possible association between phthalate exposure and endometriosis have yielded contradictory findings [40, 41]. In order to better elucidate this potential relationship, we performed a systematic review and meta-analysis of available literature. We evaluated reported levels of phthalates in women affected by and not affected by endometriosis. Furthermore, we assessed the risk of developing endometriosis in women with and without phthalate exposure.

## Materials and methods

### Protocol and registration

This study was exempt from institutional review board approval because it did not involve human subjects. Study conduct adhered to the Preferred Reporting Items for Systematic Reviews and Meta-Analyses (PRISMA) guidelines [42] and the corresponding checklist is provided in the Supplementary Material. The study protocol was registered with PROSPERO (ID: CRD42017083351) before initiating the review process.

### Eligibility criteria

The selection criteria were structured in accordance with the Patients, Intervention, Comparison, and Outcomes (PICO) model. In detail, we assessed phthalate exposure in women with endometriosis and control subjects. Control subjects were defined as women without endometriosis as determined by imaging or surgical evaluation.

### Search strategy

We conducted a systematic search of the MEDLINE (PubMed), SCOPUS, and ISI WEB OF SCIENCE databases to identify all relevant studies published prior to November 1, 2020. Combinations of the following keywords and MESH search terms were used: (“*phthalic acid”* OR *“phthalate”* OR *phthalate metabolites”*) AND (*“endometriosis”* OR *“endometrioses”* OR *“endometrioma”* OR *“endometriomas”*)*.* Eligible studies were clinical studies (prospective or retrospective) of women with endometriosis that reported urinary or blood levels of any known phthalate compound or metabolite and were published in a peer-reviewed journal. Case series, case reports, book chapters, congress abstracts, and grey literature were not included. No date or language restrictions were adopted and queries were limited to human studies. The bibliographies of relevant reviews and articles were hand-searched to complement the database search.

### Study selection

Two reviewers (AC, FC) independently screened the titles and abstracts of eligible studies. Duplications were removed manually and using Endnote online software. Full-text manuscripts were retrieved to confirm eligibility. Disagreements were resolved by discussion among the authors and, if required, with the involvement of the most experienced authors (PC, CA).

### Data extraction

Demographic variables of interest included age and the presence of endometriosis. Outcome variables of interest included any reported measurement of phthalates and phthalate metabolites in blood or urine as listed in Table 1. Data were extracted independently by two reviewers (AC, LC) using a data extraction sheet adapted from the Cochrane data extraction template for non-randomized controlled trials (https://dplp.cochrane.org/data-extraction-forms). Disagreements were resolved by discussion with the senior authors (PC, CA). In cases of missing data, the authors were contacted by email.

**Table 1:** Features of the included studies

Author, year, (ref)	Study design	Population		Endometriosis diagnosis	Methods	Sample	Phthalates	Confounders adjusted for	Conclusions	NOS score
		Country	Cases/controls (n)							
Cobellis et al., 2003	Case-control	Italy	35/24	Laparoscopy and histology	HPLC	Blood Peritoneal fluid	DEHP, MEHP.	/	Significantly higher serum levels of DEHP in patients with endometriosis.	7
Reddy et al., 2006^a^	Prospective Case- control	India	49/59	Laparoscopy	GC	Blood	DnBP, DEHP, DnOP, BBP.	/	Significantly higher serum levels of DnBP, DEHP, DnOP, and BBP in women with endometriosis compared to controls of infertile or fertile gynecological patients.	7
Reddy et al., 2006^b^	Case-control	India	85/135	Laparoscopy	GC	Blood	DnBP, BBP, DnOP, DEHP.	/	DnBP, BBP, DnOP, and DEHP were higher in endometriosis groups and significantly correlated with endometriosis severity.	6
Rozati et al., 2008	Prospective Case-control	India	99/135	Laparoscopy	HPLC	Blood	DMP, DEP, DnBP, BBP. DEHP.	/	Significantly higher serum levels of DMP, DEP, DnBP, BBP, and BEHP in women with endometriosis and correlated with endometriosis severity.	5
Huang et al., 2010	Case-control	Taiwan	28/29 16 adenomyosis	Laparotomy and histology	HPLC MS	Urine	MMP, MEP, MnBP, MBzP, MEHP, MEOHP, MEHHP.	Creatinine	Higher levels of MnBP in women with endometriosis.	7
Weuve et al., 2010	Cross-sectional	USA	87/1020	Questionnaire	HPLC MS	Urine	MEHP, MBP, MEP, MBzP, MEHHP, MEOHP.	Age Ethnicity Age at menarche Pregnancy Breastfeeding Creatinine	Positive associations for MBP and inverse associations for MEHP in relation to endometriosis.	5
Kim et al., 2011	Prospective Case-control	Korea	97/169	Surgery and histology	HPLC MS	Blood	MEHP, DEHP.	Pregnancy BMI	MEHP and DEHP levels were higher in endometriosis women.	8
Upson et al. 2013	Case-control	USA	92/195	Surgery	HPLC MS	Urine	MEHP, MEHHP, MEOHP, MECPP, MBzP, MEP, MiBP, MnBP.	Age Education Smoking Reference year Alcohol Creatinine	Strong inverse association between endometriosis risk and urinary concentration of MEHP, accompanied by the suggestion of weaker inverse associations with urinary concentrations of other DEHP metabolites, as MEHHP and MEOHP, and ΣDEHP. Urinary concentrations of MBzP and MEP may be associated with increased risk of endometriosis.	7
Buck Louis et al., 2013	Matched cohort	USA	190/283	Surgery and histology (study group) MRI (control group)	ED-SPE MS	Urine	MECPP, MCMHP, MEOHP, MEHHP, MEHP, MCPP, MMP, MEP, MiBP, MCHP, MBzP, MNP, MOP, MBP, BPA.	Age BMI Creatinine	In the population cohort, six phthalate metabolites (MBP, MCMHP, MECPP, MEHP, MEHHP, and MEOHP) were significantly associated with approximately a two-fold increase in the odds of an endometriosis diagnosis. Two phthalates were associated with endometriosis in the operative cohort when restricting to visualized and histologic endometriosis (MOP), or when restricting comparison women to those with a postoperative diagnosis of a normal pelvis (MEHP).	8
Kim et al., 2015	Prospective Case-control	Korea	55/33	Surgery and histology	HPLC MS	Urine	MEHHP, MEOHP, MnBP, MBzP, MECPP.	Age Deliveries Creatinine	Log-transformed urinary concentration of MEHHP, MEOHP, and MECPP was significantly higher in women with endometriosis compared with controls.	8
Sun et al., 2016	Case-control	China	134/176	Surgery and histology	GC	Blood	DEP, DEHP, DnBP.	/	Higher DEHP and DnBP in women with endometriosis.	7
			133/158		HPLC MS	Urine	MMP, MEP, MiBP, MnBP, MEHP, MEOHP, MEHHP, MECPP, MCMHP.	Creatinine	Higher MEHP in women with endometriosis. Lower MEP, MiBP, MnBP, MEOHP, and MEHHP in women with endometriosis.	
Pednekar et al., 2018	Case-control	India	11/34	Not defined	GC MS	Blood	BPA, MMP, MBzP, MEHP, MEHHP.	/	Higher BPA, MBzP, and MEHHP in women with endometriosis. No difference in MMP and MEHP levels.	5
Nazir et al., 2018	Case-control	Pakistan	50/50	Laparoscopy	HPLC	Blood	DEHP	/	DEHP was not found in controls. Comparison of DEHP among stages of endometriosis revealed an increasing trend with advanced stages (III and IV).	6
Moreira Fernandez et al., 2019	Case-control	Brazil	30/22	Surgery and histology	HF-LPME GC-MS	Urine	BPA, MMP, MiBP, MBP, MCHP, MiNP, MOP, MBzP, MEHP.	Creatinine	No association between phthalate metabolites and endometriosis.	6

*NOS*, Newcastle Ottawa Scale; *HPLC*, high-performance liquid chromatography; *GC*, gas chromatography; *MS*, mass spectrometry; *ED-SPE*, enzymatic deconjugation followed by solid phase extraction; *HF-LPME*, hollow fiber liquid phase microextraction; *MRI*, magnetic resonance imaging. The definitions of phthalates are found in the “Glossary of phthalates (alphabetic order)” and “Glossary of phthalate metabolites” sections

### Risk of bias and quality assessment

Two authors (IS, FC) independently assessed the risk of bias and quality of included studies using the Newcastle-Ottawa Scale (NOS) [43]. NOS scores were adjudicated in accordance with three data quality issues: selection of the study group, comparability between groups, and how the exposed/unexposed cohorts were identified. Disagreements were resolved by discussion with the senior authors (PC, CA).

### Outcomes

The primary outcome was phthalate levels in blood and/or urine. An overall odds ratio (OR) and 95% confidence interval (CI) was calculated to assess the relationship between phthalate exposure and the development of endometriosis.

### Statistical analysis

Phthalate levels derived from individual studies were converted to parts per million in blood and urine (creatinine-adjusted). In order to assess standardized mean differences (SMDs), means and standard deviations were either recorded directly or, when the number of subjects was known, estimated from the median and range/interquartile range using published methods [44, 45]. In some cases, standard deviations were calculated from confidence intervals (when the mean and number of subjects were known). The Higgins method [46] was used to transform geometric means into arithmetic means based on the relationship between raw and log-transformed measurements. In a conservative approach, the random effects estimate of SMD (and relative 95% CI), which allow for variation of true effects across studies, were taken as main results. Furthermore, compounds were compared, when possible, also meta-analyzing adjusted OR and relative CI. We quantified heterogeneity using the I^2^ statistic, which describes the percentage of total variation across studies attributable to heterogeneity rather than chance (I^2^ values of 25%, 50%, and 75% correspond to cut-off points for low, moderate, and high degrees of heterogeneity). Each compound was investigated as a different group in order to increase the specificity of the analysis and avoid further distortions. Meta-analyses were performed on a minimum of three studies; otherwise, results were reported as qualitative. All analyses were performed using STATA version 16.0 (StataCorp 2019, Stata Statistical Software: Release 16. College Station, TX: StataCorp LLC).

## Results

### Study selection, characteristics, and risk of bias within studies

**Fig. 1:**
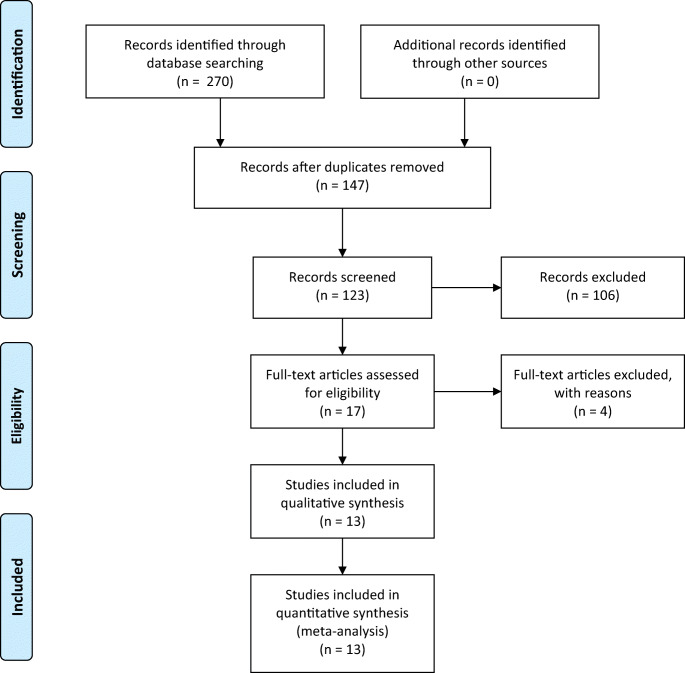
PRISMA flow chart diagram for study selection

A total of 270 articles were initially identified by the search (PubMed, 32; ISI Web of Knowledge, 71; Scopus, 72; Embase 95); of these, 147 articles were duplications and thus removed. The titles and abstracts of 123 articles were scrutinized and ultimately 18 were selected for full text retrieval and eligibility assessment. Four papers were excluded for the following reasons: two papers did not meet the inclusion criteria [47, 48]; Huang et al. were excluded because adenomyosis and endometriosis were merged in the same group [49] and another paper was excluded because the control group included women with stage I endometriosis [50]. Thus, 14 articles [40, 41, 51–62] were included in the quantitative and qualitative analyses (Figure 1). Noteworthy, Buck Louis et al. [57] included operative and population cohorts that were counted as 2 separate studies for the analysis of urinary compounds. The characteristics of included studies and risk of bias are reported in Table 1.

**Fig. 2:**
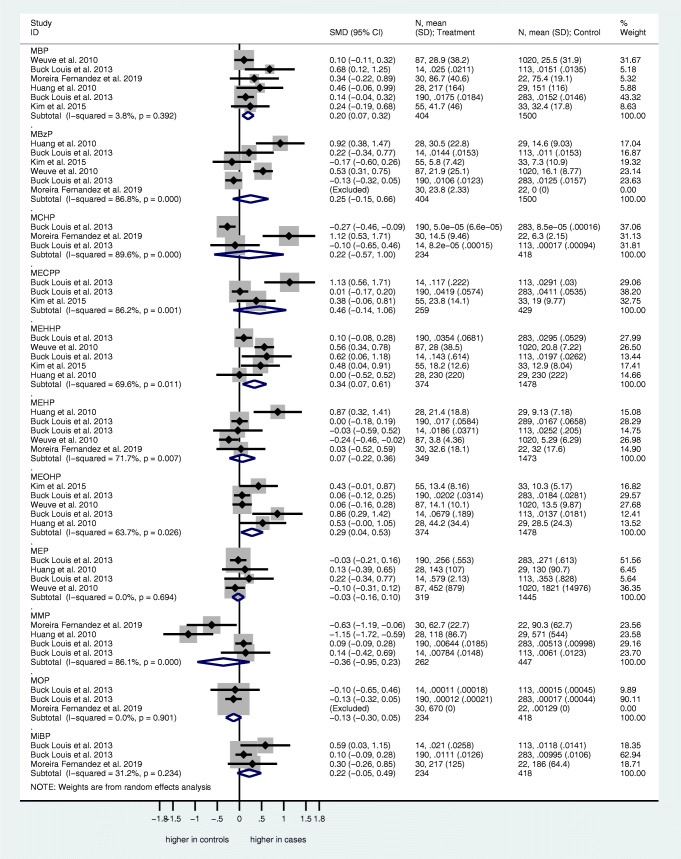
Forest plots for the association of urine phthalate concentrations and endometriosis. Forest plots of urine phthalate concentrations in case and control subjects. For each study, standardized mean differences (SMD) and 95% confidence intervals (95% CIs) are denoted by black diamonds and black lines, respectively. Grey boxes are inversely proportional to study weight. The combined SMD estimate for all subtypes is represented by a blue diamond, where diamond width corresponds with the 95% CI bounds. Furthermore, n-values, means, and standard deviations for cases and control subjects are shown for each study. The p-value for heterogeneity (P-het) of SMDs and I^2^ value are shown

**Fig. 3:**
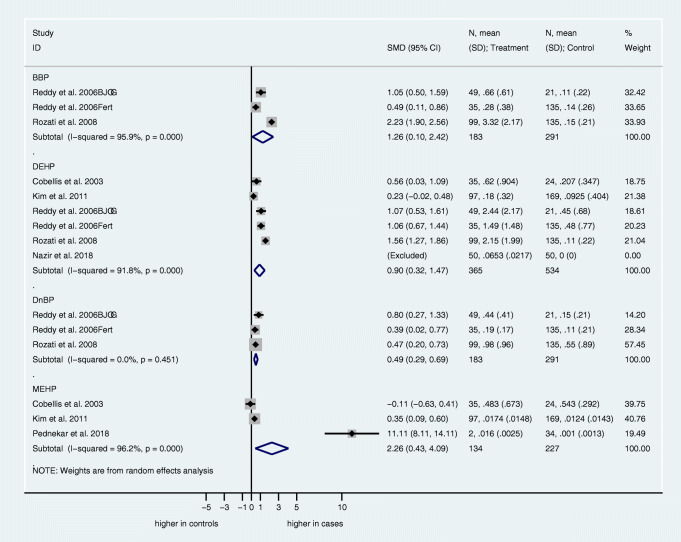
Forest plots for the association of blood concentrations of phthalates and endometriosis. Forest plots of blood phthalate concentrations in case and control subjects. For each study, standardized mean differences (SMD) and 95% confidence intervals (95% CIs) are denoted by black diamonds and black lines, respectively. Grey boxes are inversely proportional to study weight. The combined SMD estimate for all subtypes is represented by a blue diamond, where diamond width corresponds with the 95% CI bounds. Furthermore, n-values, means, and standard deviations for cases and control subjects are shown for each study. The p-value for heterogeneity (P-het) of SMDs and I^2^ value are shown

Quantitative assessment of data was carried out for the following phthalate compounds:

- Urinary: MBzP (mono-benzyl phthalate), MECPP (mono-[2-ethyl-5-carboxypentyl] phthalate), MBP, MCHP (mono-cyclohexyl phthalate), MEOHP, MEHHP, MMP (mono-methyl phthalate), MOP (mono-octyl phthalate), MEP, MEHP, MiBP (Figure 2).
- Blood: BBP (butyl-benzyl phthalate), DEHP, DnBP (di-*n*-butyl phthalate), MEHP (Figure 3).

A qualitative assessment was performed for the remaining compounds, which were overall analyzed by less than 3 studies:

- Urinary: ∑DEHP = MEHP + MEHHP + MEOHP + MECPP + MCMHP (mono-[(2-carboxymethyl)-hexyl] phthalate).
- Blood: DnOP (di-*n*-octyl phthalate), DMP (di-methyl phthalate), DEP (di-ethyl phthalate), MCPP (mono-[3-carboxypropyl] phthalate), MNP (mono-*iso*-noyl phthalate), MCMHP, MiNP (mono-*iso*-nonyl phthalate).

**Fig. 4:**
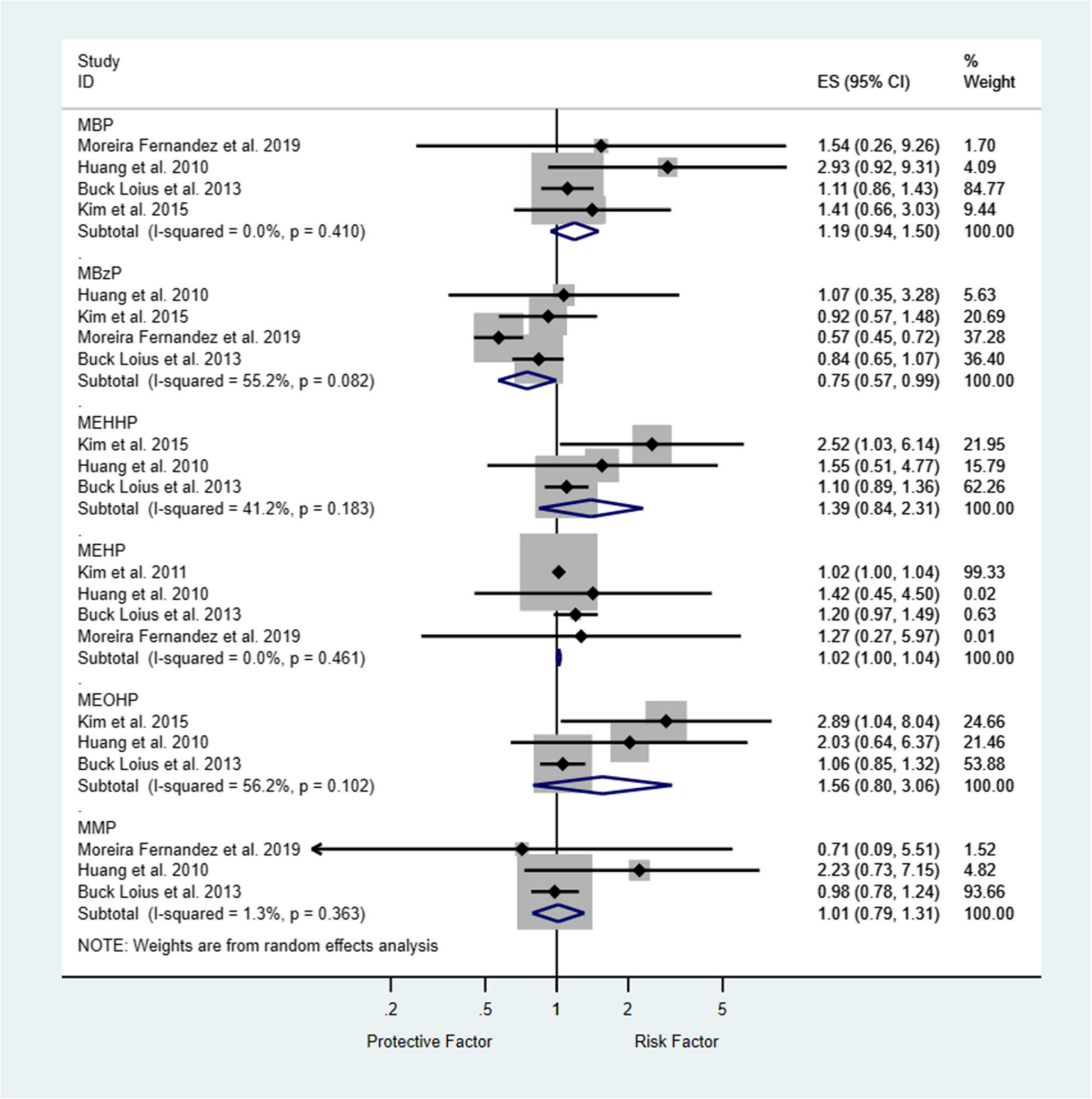
Odds ratios for the risk of endometriosis. Forest plots for phthalate exposure and the development of endometriosis. For each study, odds ratios (ORs) and 95% confidence intervals (95% CIs) are denoted by black diamonds and black lines, respectively. Grey boxes are inversely proportional to study weight. The combined OR estimate for all subtypes is represented by a blue diamond, where diamond width corresponds with the 95% CI bounds. MMP data from Moreira Fernandez et al. [58] has a very low weight and a wide confidence interval, such that the lower interval is represented with a single-headed arrow. The p-value for heterogeneity (P-het) of ORs and I^2^ are shown

In addition, ORs for endometriosis were calculated for the following phthalates:

- MBzP, MEHHP, MEHP, MEOHP, MMP, MBP (Figure 4).

For some of the abovementioned phthalates, it was not possible to consider all available studies in the quantitative analysis due to   incomparability of the study data. In such cases, data were listed in as individual evidence supporting or contrasting the quantitative analysis. The results of Sun et al. [59] were evaluated only qualitatively due to the absence of variability measurement (e.g., standard deviations). The quantitative OR analysis only included studies in which there was a comparison between women with and without endometriosis [55, 57, 59, 62]. ORs from Upson et al. [41] and Weuve et al. [54] were only evaluated qualitatively since the comparison was between different quartiles of exposure (i.e., first vs. fourth quartiles).

### Synthesis of the results

#### Urinary phthalates: quantitative analysis

Urinary phthalate concentrations were quantified in five studies [54, 55, 57, 58, 62]. The concentrations of MBzP, MECPP, MCHP, MEHP, MiBP, MMP, MOP, and MEP were comparable between the endometriosis and control groups. In contrast, urine concentrations of MBP (SMD 0.20, 95% CI 0.07–0.32; p <0.05), MEOHP (SMD 0.29, 95% CI 0.04–0.53; p <0.05), and MEHHP (SMD 0.34, 95% CI 0.07–0.61; p <0.05) were significantly higher in the endometriosis group compared to the control group (Figure 2).

#### Urinary phthalates: qualitative analysis

Two studies assessed urinary levels of MCMHP [57, 59]. In the population cohort reported by Buck Louis et al. [57], women with endometriosis diagnosed by magnetic resonance imaging had higher urinary concentrations of MCMHP (endometriosis vs. control, geometric mean [95% CI]; 53.5 [25.9–110.50] ng/ml vs. 22.5 [19–26.6] ng/ml; p <0.05) and MCPP (5.75 [3.38–9.8] ng/ml vs. 4.06 [3.41–4.83] ng/ml; p <0.05) compared to control subjects. Sun et al. [59] reported significantly higher concentrations of urinary MEHP and MMP in the endometriosis group vs. control group but significantly higher concentrations of MBP, MECPP, MEOHP, MEHHP, MEP, and MiBP in the control group vs. endometriosis group. Furthermore, the authors observed that the sum of urinary metabolites of DEHP (∑DEHP = MEHP + MEHHP + MEOHP + MECPP + MCMHP) was significantly higher in the endometriosis group than in the control group. Moreira Fernandez et al. [62] did not observe a statistically significant difference in urinary MiNP concentration between the endometriosis and control groups.

#### Blood phthalates: quantitative analysis

Blood phthalate concentrations were quantified in seven studies [40, 51–53, 56, 60, 61]. BBP (SMD 1.26, 95% CI 0.10–2.42; p <0.05), DEHP (SMD 0.90, 95% CI 0.32–1.47; p <0.05), DnBP (SMD 0.49, 95% CI 0.29–0.69; p <0.05), and MEHP (SMD 2.26, 95% CI 0.43–4.09; p <0.05) were significantly higher in the endometriosis group compared to the control group (Figure 3).

#### Blood phthalates: qualitative analysis

Only one study assessed MBzP, MEHHP, and MMP in women with and without endometriosis [61]. MBzP and MEHHP were significantly higher in the endometriosis group than in the control group [61]. MMP concentrations were similar between groups. DMP was evaluated in one study [53] reporting significantly higher levels in controls than in women with endometriosis. Sun et al. [59] reported significantly higher blood levels of DnBP in patients with endometriosis than in control subjects. DnOP was evaluated in two studies [51, 52], both of which reported significantly higher values in women with endometriosis than in control subjects. The highest concentrations of DnOP were observed in women with stage IV endometriosis [51, 52]. Finally, two studies [53, 59] reported that DEP concentrations were significantly higher in women with endometriosis than in control subjects.

#### Risk of endometriosis: quantitative analysis

MBzP exposure was associated with a significantly lower risk of endometriosis in our analysis (OR: 0.75, 95% CI: 0.57–0.99; p <0.05). MBP, MEOHP, MEHHP, MEHP, and MMP exposure tended to increase the risk of endometriosis, but this effect was not statistically significant (Figure 4).

#### Risk of endometriosis: qualitative analysis

Upson et al. [41] observed a significant negative association between blood MEHP concentration and endometriosis risk (adjusted OR: 0.2, 95% CI: 0.08–0.6; p=0.007) in a comparison of women with exposure in the highest quartile (≥ 11.1 ng/ml) versus the lowest quartile (≤ 1.1 ng/ml). In contrast, Weuve et al. [54] observed no significant association between MEHP and endometriosis (OR: 0.44, 95% CI: 0.19–1.02) in a comparison of women with exposure in the highest quartile (≥ 6.4 ng/mg) versus the lowest quartile (≤ 1.4 ng/mg). Weuve et al. [54] and Upson et al. [41] similarly found no difference in the risk of endometriosis between women in the highest and lowest quartiles of exposure to MBzP, MBP, DEHP, MEHHP, MEOHP, and MEP.

## Discussion

### Main findings

The present meta-analysis found that women with endometriosis had higher urinary levels of MBP, MEOHP, and MEHHP and higher blood levels of BBP, DEHP, DnBP, and MEHP than women without endometriosis. Conversely, women exposed to MBzP had a significantly lower risk of developing endometriosis compared to the control group.

### Interpretation of results and clinical consideration

Endometriosis is an estrogen-related disorder with several consequences for women’s fertility and quality of life. Growing evidence implicates phthalate exposure in both the development of endometriosis and its severity [19]. In vitro studies have demonstrated that DEHP promotes endometrial cell viability [63] and endometrial tissue growth outside of the uterine cavity [58]. Specifically, endometrial cells treated in vitro with DEHP exhibit cellular invasiveness and the activation of molecular pathways involved in the establishment of endometriosis and endometrial proliferation (MMP-2 and -9 activation, Erk phosphorylation, and p21-activated kinase expression) [58]. The same study found that endometrial implant growth was accelerated in DEHP-fed mice in comparison to normally fed mice [58]. Phthalates have also been implicated in the development of endometriosis by inducing oxidative stress [21, 64]. Indeed, phthalate exposure increases the production of reactive oxygen species and at the same time decreases the expression of antioxidant factors such as superoxide dismutase and glutathione peroxidase [64]. Furthermore, phthalates exert a positive, dose-dependent effect on estrogen receptor expression [63]. The action of phthalates on estrogen receptors may also play a role in the development of the estrogen-sensitive tumors such as breast and ovarian cancers [65, 66]. The most recent Danish nationwide cohort study involving 1,12 million women at-risk for first cancer diagnosis demonstrated that high-levels of DBP exposure were associated with a two-fold increase in the risk of developing estrogen receptor-positive breast cancer (hazard ratio, 1.9; 95% CI 1.1–3.5) [65].

In our analysis, we found that specific phthalates were associated with endometriosis. MEHHP and MEOHP are two urine metabolites of DEHP; in our study, all three were evaluated in the urine and blood of women with endometriosis, respectively. Noteworthy, Buck Louis et al. [57] reported an association between the summed concentration of all DEHP metabolites (MECPP, MCMHP, MEHHP, MEOHP, and MEHP) and the development of endometriosis. DEHP-derived metabolites are widely found in cosmetic and personal care products used by women [67]. DEHP-derived metabolites have also been associated with other common reproductive disorders such as polycystic ovary syndrome (PCOS) [68], recurrent pregnancy loss [69], and even reproductive disfunction in men [70].

MBP is another metabolite of DnBP that was consistently higher among women with endometriosis in our study. Similar to DEHP and its metabolites, DnBP derivatives have also been posited to affect reproductive health [51, 53] and in one study were detected in pregnant women across all three trimesters of gestation [71]. Given the association of phthalates with spontaneous pregnancy loss [72] and possible health consequences for offspring [73], protective strategies should be adopted among women of childbearing age. These strategies potentially include healthier food choices (e.g., organic foods [74] and folic acid supplementation [75]), although the efficacy of these preventive measures requires confirmation in clinical studies.

A surprising finding of our study was a protective effect of MBzP exposure on endometriosis risk. This finding could be explained by the evidence that androgen receptors could be stimulated by MBzP [32]. The effect of androgens against endometriosis could partially explain our observation. Nonetheless, this hypothesis should be corroborated by further studies.

From a clinical perspective, our findings suggest that environmental toxicant exposure should be carefully investigated during the management of women with reproductive disorders including endometriosis. To this extent, in 2010, the World Health Organization (WHO) launched the International Program on Chemical Safety with the aim of assessing and managing the risks associated with hazardous chemical exposure. Given the robust and profound effect of toxic environmental agents on reproductive health [76], the development of special interest groups fully devoted to research in this field is necessary to better inform the issue and guide decision-making by clinicians and local health authorities. Reproductive specialists should take care to educate themselves regarding potentially harmful environmental toxicants and occupationally exposed populations.

### Strengths and limitations

This is the most updated meta-analysis to address the association between phthalate exposure and endometriosis. Our comprehensive analysis considered more than 20 phthalates, in contrast with previous reviews [77, 78]. Moreover, the analysis is strengthened by adherence to the PRISMA guidelines and registration of the study protocol with PROSPERO.

Although our study had several methodological strengths as a meta-analysis, some important limitations must be considered when interpreting our findings. A major limitation was related to the methodological weakness of the included studies. Most studies were retrospective case-control designs and some of them enrolled a very low number of participants [61, 62]. The largest study was cross-sectional [54]. The method by which patients were diagnosed with endometriosis was not always specified [61], or alternatively the diagnosis was self-reported [54] or a diagnosis of endometriosis was excluded based on self-reported fertility [60]. Despite the argument that population cohorts are more representative for association studies [41], the diagnosis of endometriosis relies on histology and laparoscopy as gold standards and therefore it appears difficult to overcome the risk of misclassification of controls in population cohorts. Furthermore, control subjects chosen on the basis of laparoscopic examination often suffer from other pathologic conditions possibly linked to EDCs exposure [78]. We hypothesize that contradictory results reported in the literature are at least partially related to these discrepancies among studies.

Another methodological issue resides in the sampling of phthalates. Some authors supposed that the use of plastic collection tubes can contaminate samples [56]. While the use of disposable glassware easily circumvents this issue, these precautions were only taken in more recent studies [60, 61]. Moreover, urinary estimation may be the best method for measuring phthalate exposure as the rapid peripheral metabolism of these compounds can complicate blood assessment [79, 80]. Another important observation is that none of the studies included in our meta-analysis took multiple urine samples to confirm the chronicity of exposure [26, 78]. Moreover, the timing of sample collection often varied (immediately before or after surgery) and samples taken after surgery may have been contaminated as the result of intravenous therapies or the laparoscopic procedure itself [54, 81].

Several included studies failed to rigorously control for possible confounding factors [40, 51–53, 59–61]. For example, participants should have been asked about and screened for medications associated with possible phthalate contamination (e.g., in pill coatings) [27]. The selection of the control cohort was also different among trials, and only two studies used matched cohorts [40, 57]. Unfortunately, we also excluded two large studies from our quantitative analysis (Upson et al. [41] and Weuve et al. [54]) in which the risk of endometriosis was evaluated in different quartiles of exposure rather than against a control group. Finally, another limitation is that we were unable to evaluate the relevance of phthalate exposure to different stages of endometriosis, considering that very few studies have compared levels of phthalate exposure among different stages of the disease.

## Conclusions

Our findings showed a possible association between exposure to some phthalates and endometriosis. Our results should be interpreted with caution given the intrinsic methodological limitations and heterogeneity of included studies. Indeed, most of the studies were retrospective with low numbers of participants and different methods were applied for assessing phthalate exposure. Nonetheless, the quantity of evidence on this topic suggests that more robust investigations are necessary to ascertain a link between phthalate exposure and endometriosis and translate these findings into clinical practice.

## Supplementary Information

ESM 1 (PDF 115 kb)
